# Defect‐Engineered LiYO_2_ Interlayers Enabling Fast Interfacial Li^+^ Transport in All‐Solid‐State Batteries

**DOI:** 10.1002/advs.77729

**Published:** 2026-09-10

**Authors:** Sodam Kim, Yun Seong Byeon, Jaewoo Jung, Mingyu Cho, Hyunmin Lee, Jung Ho Kim, Min‐Sik Park

**Affiliations:** ^1^ Department of Materials Science and Engineering Kyung Hee University Giheung‐gu, Yongin Republic of Korea; ^2^ Institute For Superconducting & Electronic Materials (ISEM) Australian Institute for Innovative Materials (AIIM) University of Wollongong North Wollongong New South Wales Australia

**Keywords:** aliovalent substitution, all‐solid‐state batteries, interfacial stability, LiYO_2_ coating, Ni‐rich layered oxide cathode

## Abstract

All‐solid‐state batteries (ASSBs) are desirable for next‐generation energy storage systems due to their high energy density and enhanced safety. However, chemical and electrochemical incompatibility between Ni‐rich layered oxide cathodes and sulfide solid electrolytes, particularly Li_6_PS_5_Cl (LPSCl), limits practical applications. Direct contact between these components accelerates oxidative decomposition of LPSCl, forming resistive interphase products, sluggish interfacial Li^+^ transport, and rapid capacity decay. Here, we propose defect‐engineered LiYO_2_ (LYO) coatings as chemically stable and conductive interlayers for Ni‐rich cathodes. To improve the intrinsically limited Li^+^ transport of LYO, aliovalent substitution is employed based on Li^+^ defect chemistry. Zn^2+^‐substituted LYO (LYZnO, Li_1.05_Y_0.95_Zn_0.05_O_2_) and Zr^4+^‐substituted LYO (LYZrO, Li_0.95_Y_0.95_Zr_0.05_O_2_) are designed to introduce Li‐excess and Li‐vacancy defects, respectively. Importantly, both configurations successfully maintain the chemically robust Y–O polyhedral framework of LYO. When applied as nanoscale coatings on Ni‐rich cathode particles, both LYZnO and LYZrO effectively suppress parasitic decomposition of LPSCl and improve Li^+^ transport across the cathode–electrolyte interface. Li‐vacancy‐type LYZrO exhibits superior interfacial stabilization compared with Li‐excess‐type LYZnO, owing to more favorable vacancy‐mediated Li^+^ migration. These findings demonstrate that defect engineering of stable oxide coatings is an effective strategy to construct reliable and kinetically favorable cathode interfaces in ASSBs.

## Graphical abstract

Defect‐engineered LiYO_2_ interlayers are designed through aliovalent Zn^2+^ and Zr^4+^ substitution to regulate Li^+^ point‐defect chemistry in Ni‐rich cathodes. Compared with Li‐excess‐type LYZnO, Li‐vacancy‐type LYZrO enables faster Li^+^ transport, suppresses Li_6_PS_5_Cl decomposition, lowers interfacial resistance, and mitigates polarization growth, thereby stabilizing cathode–sulfide electrolyte interfaces in all‐solid‐state batteries.

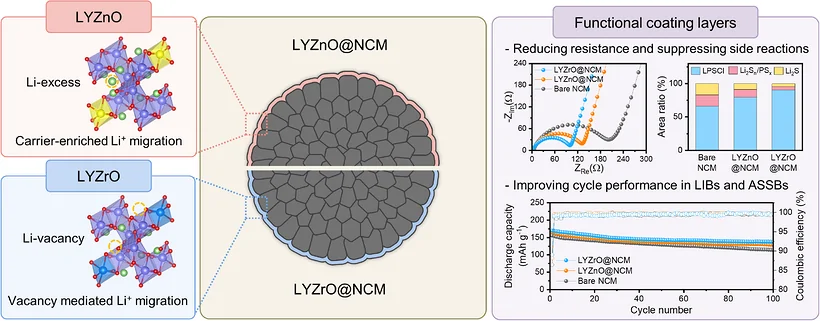

## 1 Introduction

All‐solid‐state batteries (ASSBs) based on sulfide solid electrolytes have attracted considerable attention because of their potential to achieve both high energy density and enhanced safety [1, 2, 3, 4, 5, 6]. Among various sulfide electrolytes, argyrodite Li_6_PS_5_Cl (LPSCl) is particularly attractive owing to its high ionic conductivity, on the order of 10^−3^ S cm^−1^, and favorable mechanical deformability, which enables intimate solid–solid contact within composite cathodes [7, 8, 9]. Despite these advantages, the chemical and electrochemical incompatibility between Ni‐rich layered oxide cathodes and LPSCl remains a major bottleneck for the practical operation of sulfide‐based ASSBs [10, 11]. Direct contact between Ni‐rich cathode and LPSCl solid electrolyte at high operating voltages promotes the oxidative decomposition of the LPSCl, leading to the formation of resistive sulfur‐ and phosphorus‐containing interphase products [12, 13, 14]. The accumulation of these decomposition products increases interfacial resistance, hinders Li^+^ transport across the cathode–electrolyte interface, and accelerates capacity decay [15, 16]. Therefore, stabilizing the Ni‐rich cathodes–LPSCl interface is essential for achieving reliable ASSB performance [17].

Surface modification has proven broadly effective for regulating interfacial Li^+^ transport kinetics in electrode materials. Among these approaches, surface coating of cathode particles has been widely investigated to mitigate interfacial degradation in ASSBs [18, 19, 20, 21]. Conventional oxide coating materials, such as Li_2_ZrO_3_, LiNbO_3_, Li_3_PO_4_, and Al_2_O_3_, can physically isolate the cathode particles from sulfide electrolyte and suppress parasitic side reactions [22, 23, 24, 25, 26, 27, 28]. However, many of these oxide coatings possess insufficient Li^+^ conductivity, which can impose an additional Li^+^ transport barrier and increase interfacial overpotential despite their chemical passivation effect [29, 30]. Thus, an ideal protective interlayer should simultaneously provide chemical protection against LPSCl decomposition and enable efficient Li^+^ transport across the solidsolid interface [31].

LiYO_2_ (LYO) is a promising candidate for Ni‐rich cathode–electrolyte interface because it combines chemical robustness with Li^+^‐conducting capability [32, 33]. LYO crystallizes in a monoclinic structure with the P*2_1_/c* space group, featuring a three‐dimensional oxide framework composed of Li–O and Y–O polyhedra [34]. In this framework, Y^3+^ form distorted YO_6_ octahedra connected through edge‐ and corner‐sharing configurations, while Li^+^ occupy oxygen‐coordinated sites within the oxide network. The chemically stable Y–O framework can contribute to interfacial protection, whereas the connected Li–O coordination environments can provide pathways for Li^+^ migration. However, Li^+^ conduction in LYO is governed by thermally activated migration between neighboring Li sites and is therefore strongly dependent on the concentration of mobile defects and available vacant sites [35, 36]. When the number of accessible Li vacancies is insufficient, the intrinsic ionic conductivity of LYO can be limited, potentially limiting its effectiveness as a protective interlayer.

Aliovalent cation substitution offers a rational approach to regulating Li^+^ defect chemistry and enhancing Li^+^ transport kinetics within the LYO framework [35, 36]. Substitution of Y^3+^ with tetravalent Zr^4+^ introduces charge‐compensating Li vacancies, thereby increasing the number of accessible vacant sites for Li^+^ migration and promoting vacancy‐mediated Li^+^ migration [35]. On the other hand, substitution of Y^3+^ with divalent Zn^2+^ induces Li‐excess‐type defect chemistry by increasing the Li^+^ content in the host framework [36]. Although both strategies can modify the local Li^+^ transport environment, Li‐vacancy‐type defect engineering is expected to provide a more direct and efficient migration pathway by lowering the activation energy barrier for Li^+^ migration through vacant sites.

Based on these theoretical and structural considerations, Zn^2+^‐substituted LYO (LYZnO, Li_1.05_Y_0.95_Zn_0.05_O_2_) and Zr^4+^‐substituted LYO (LYZrO, Li_0.95_Y_0.95_Zr_0.05_O_2_) were designed to compare the effects of Li‐excess and Li‐vacancy defect chemistries on the Li^+^ transport properties of the LYO framework. These defect‐engineered LYO were further applied as nanoscale coatings on Ni‐rich cathode particles to stabilize the cathode–LPSCl interface in ASSBs. Combined electrochemical, structural, and post‐mortem analyses revealed that aliovalent defect engineering of LYO enhances interfacial Li^+^ transport, suppresses parasitic decomposition of LPSCl, and improves the structural stability of Ni‐rich cathodes. In particular, the Li‐vacancy‐type LYZrO provides more effective interfacial stabilization than Li‐excess‐type LYZnO, demonstrating that vacancy‐mediated defect engineering is a promising strategy for designing reliable and ion‐conductive oxide coatings for ASSBs.

## 2 Results and Discussion

### 2.1 Aliovalent Regulation of Li^+^ Defect Chemistry in monoclinic LiYO_2_

**FIGURE 1:**
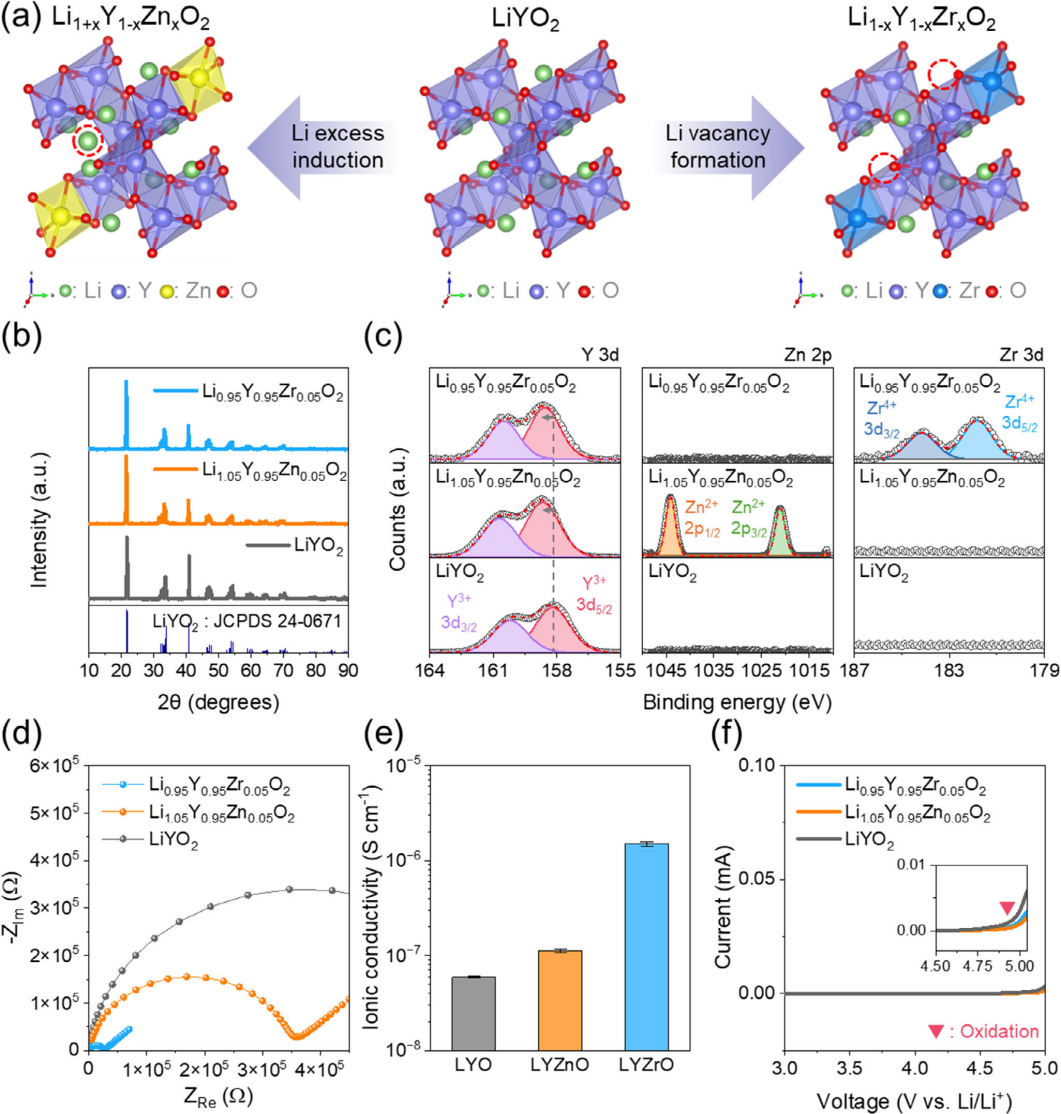
Schematics of (a) LiYO_2_ crystal structure (monoclinic) and Zn, Zr elemental substitution on LiYO_2_ crystal structure. (b) XRD patterns, (c) XPS Y 3d, Zn 2p and Zr 3d spectra, (d) Nyquist plots, (e) Ionic conductivity and (f) Linear sweep voltammetry (LSV) curves of LYO, LYZnO and LYZrO.

The structural design of defect‐engineered LiYO_2_ (LYO) through aliovalent substitution is illustrated in Figure 1a. Monoclinic LYO was selected as a common oxide host because of its favorable physicochemical properties, including Li^+^‐conducting capability and excellent chemical stability [32, 33]. LYO crystallizes in the P*2_1_/c* space group and consists of interconnected YO_6_ octahedra and oxygen‐coordinated Li sites [34]. In this framework, distorted YO_6_ units form a robust three‐dimensional oxide backbone through edge‐ and corner‐sharing configurations, while Li^+^ are distributed over oxygen‐coordinated sites throughout the structure. Because Li^+^ conduction in LYO occurs through migration between neighboring Li sites, its ionic conductivity is strongly influenced by the concentration of mobile defects and accessible vacant sites [35, 36]. Therefore, controlling Li^+^ point‐defect chemistry is a rational approach to enhancing Li^+^ transport within the LYO structure.

To investigate this effect, Zn^2+^ and Zr^4+^ were selected as aliovalent substituents for Y^3+^ because their ionic radii, 0.074 and 0.072 nm, respectively, are smaller than but still comparable to that of Y^3+^ (0.090 nm), allowing their incorporation into the LYO lattice without severe disruption of the host framework [37]. From a charge‐compensation perspective, substitution of Y^3+^ with divalent Zn^2+^ requires additional Li^+^ to maintain charge neutrality, resulting in Li‐excess‐type Li_1.05_Y_0.95_Zn_0.05_O_2_ (LYZnO). In contrast, substitution with tetravalent Zr^4+^ induces Li deficiency, forming Li‐vacancy‐type Li_0.95_Y_0.95_Zr_0.05_O_2_ (LYZrO). This paired design enables a direct comparison of Li‐excess and Li‐vacancy defect chemistries within the same LYO host structure by creating distinct charge‐compensation environments.

The crystal structures of LYO, LYZnO, and LYZrO were examined by powder X‐ray diffraction (XRD), as shown in Figure 1b. All diffraction peaks of LYZnO and LYZrO were well indexed to monoclinic LYO (JCPDS No. 24–0671), with no detectable crystalline impurity phases, indicating that the host LYO structure was retained after aliovalent Zn^2+^ and Zr^4+^ substitution. However, because XRD is intrinsically insensitive to quantitative Li‐site occupancy in Y‐containing oxide lattices, inductively coupled plasma mass spectrometry (ICP‐MS) was further conducted to verify the intended bulk compositions (Figure S1). Pristine LYO showed Li and Y fractions of 49.98 and 50.02 at%, respectively, consistent with the stoichiometric Li ratio of LiYO_2_. For LYZnO, the measured Li/Y/Zn ratio was 51.21/46.33/2.46 at%, which closely matches the nominal composition of Li_1.05_Y_0.95_Zn_0.05_O_2_. This result supports the successful formation of a Li‐excess composition. In contrast, LYZrO exhibited a Li/Y/Zr ratio of 48.69/48.69/2.62 at%, in good agreement with the nominal composition of Li_0.95_Y_0.95_Zr_0.05_O_2_, confirming the formation of a Li‐deficient composition. These results provide direct compositional evidence that the intended Li‐excess and Li‐deficient stoichiometries were successfully achieved in LYZnO and LYZrO, respectively. Rietveld refinement using ICP‐MS‐informed occupancy constraints revealed opposite unit‐cell volume changes for LYZnO and LYZrO relative to pristine LYO (Figure S2 and Table S1). In particular, the expanded unit‐cell volume of LYZrO, together with the stoichiometric Li/Y ratio of pristine LYO, is consistent with the proposed Li‐deficient defect chemistry rather than simple Li loss during calcination (Note S1).

The chemical states of the constituent elements were further analyzed by X‐ray photoelectron spectroscopy (XPS), as shown in Figure 1c. The Y 3d spectra of all samples exhibited characteristic Y^3+^ signals, confirming that the trivalent oxidation state of the Y host cation was preserved after aliovalent substitution. However, compared with pristine LYO, the Y 3d peaks of LYZnO and LYZrO shifted slightly toward higher binding energies, indicating that aliovalent substitution modifies the local electronic environment of the Y–O framework without changing the formal oxidation state of Y. This shift can be attributed to charge‐compensation‐driven rearrangement of the Li–O–Y coordination network, involving Li‐excess formation in LYZnO and Li‐vacancy formation in LYZrO. In addition, the Zn 2p spectrum of LYZnO showed Zn^2+^ 2p_3/2_ and 2p_1/2_ signals at 1021.1 and 1044.1 eV, respectively, while the Zr 3d spectrum of LYZrO displayed characteristic Zr^4+^ 3d_5/2_ and 3d_3/2_ signals at 181.9 and 185.3 eV, respectively [38]. These results verify the presence of Zn^2+^ and Zr^4+^ in the LYZnO and LYZrO without altering the oxidation state of the Y^3+^ host cation. Overall, structural and chemical analyses support the successful synthesis of LYZnO and LYZrO, where Zn^2+^ substitution induces Li‐excess‐type defect chemistry and Zr^4+^ substitution induces Li‐vacancy‐type defect chemistry through charge compensation. The Raman spectra further support the defect‐chemistry‐dependent modulation of the LYO framework (Figure S3). Compared with pristine LYO, the A_g_ and B_g_ vibrational modes of LYZnO shifted toward lower wavenumbers, indicating phonon softening of the local oxide framework [39, 40]. This red shift can be attributed to Zn^2+^‐induced Li‐excess defect chemistry, which modifies the Li–O–Y coordination environment and weakens the local restoring force of the lattice vibrations. In contrast, the A_g_ and B_g_ modes of LYZrO shifted toward higher wavenumbers, suggesting phonon hardening of the LYO framework. The blue shift is likely associated with Zr^4+^‐induced Li‐vacancy formation and stronger local Zr–O interactions, which render the oxide framework more rigid. These opposite Raman shifts indicate that Zn^2+^ and Zr^4+^ substitution regulate the local lattice dynamics of LYO in different ways, consistent with their distinct Li‐excess and Li‐vacancy defect chemistries.

The effect of Li^+^ defect chemistry on Li^+^ transport properties of LYO was evaluated using Nyquist plots (Figure 1d), and the corresponding ionic conductivities are summarized in Figure 1e. The pellet thickness, relative density, total resistance, and calculated room‐temperature ionic conductivity values used for this comparison are provided in Table S2. Pristine LYO exhibited an ionic conductivity of ≈6.0 × 10^−8^ S cm^−1^, whereas both LYZnO and LYZrO showed improved Li^+^ transport properties. In practice, LYZnO delivered a higher ionic conductivity of ≈1.1 × 10^−7^ S cm^−1^, suggesting that Zn^2+^‐induced Li‐excess chemistry can modify the local Li^+^ transport environment within the LYO structure. More importantly, LYZrO exhibited the highest ionic conductivity of ≈1.5 × 10^−6^ S cm^−1^, which is more than one order of magnitude higher than that of pristine LYO. This noticeable enhancement is attributed to vacancy‐mediated Li^+^ migration, where Zr^4+^‐induced Li vacancies provide accessible vacant sites for Li^+^ migration. These results demonstrate that Li‐vacancy‐type defect chemistry is more effective than Li‐excess‐type defect chemistry in promoting Li^+^ conduction within the LYO framework. Temperature‐dependent impedance measurements further quantified the ionic transport kinetics (Figure S4 and Table S2). The activation energy for ionic conduction decreased from 0.45 eV for LYO to 0.43 eV for LYZnO and 0.39 eV for LYZrO, supporting facilitated Li^+^ transport associated with the Zr^4+^‐induced Li‐deficient defect chemistry. Together with the Li‐vacancy formation supported by the occupancy‐constrained refinement, the reduced activation energy of LYZrO provides experimental evidence for vacancy‐mediated Li^+^ migration within the LYO framework.

The electrochemical stability of the samples was further evaluated by linear sweep voltammetry (LSV), as shown in Figure 1f. All samples exhibited negligible oxidative currents in the high‐voltage region relevant to NCM cathode operation, indicating sufficient oxidative stability for surface coatings. In practice, LYZnO and LYZrO maintained stable oxidative behavior up to ≈4.9 and 4.85 V vs. Li/Li^+^, respectively, demonstrating that Zn^2+^ and Zr^4+^ substitution did not compromise the electrochemical stability of the LYO framework. Taken together, these results show that both LYZnO and LYZrO simultaneously provide enhanced Li^+^ conductivity and high‐voltage stability, making them promising nanoscale coating materials for stabilizing the NCM–LPSCl interface in ASSBs. The thermally aged LYZnO‐LPSCl and LYZrO‐LPSCl mixtures showed no additional crystalline phases or new S 2p and P 2p components relative to pristine LPSCl (Figure S5), supporting their chemical compatibility under the tested conditions.

### 2.2 Surface Coating of Defect‐Engineered LiYO_2_ Materials

**FIGURE 2:**
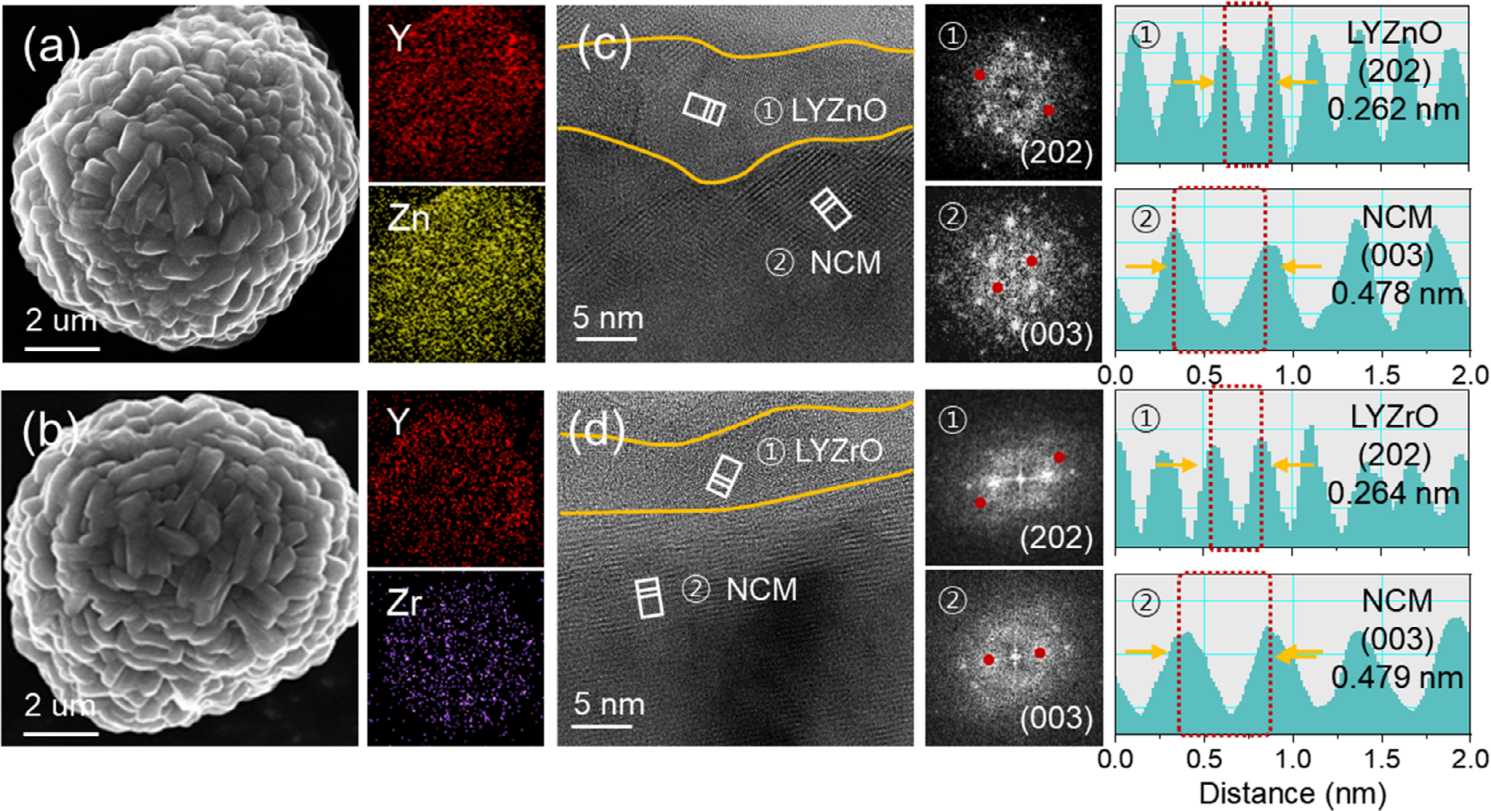
SEM images of (a) LYZnO@NCM, (b) LYZrO@NCM with EDS elemental mapping images of Y, Zn, Zr on the right side. HRTEM image with a lattice spacings and FFT patterns of the surface region on (c) LYZnO@NCM and (d) LYZrO@NCM.

To evaluate the feasibility of LYZnO and LYZrO as interlayers for Ni‐rich cathodes in ASSBs, LYZnO@NCM and LYZrO@NCM were prepared through a wet‐coating process followed by calcination. The coating process was first examined by preparing LYZnO@NCM and LYZrO@NCM with different coating contents of 0.3, 0.5, and 1.0 wt%. As summarized in Figure S6, SEM images, EDS elemental mappings, and XRD patterns confirmed that the wet‐coating and calcination processes did not induce noticeable particle collapse, severe surface damage, or impurity‐phase formation over the investigated coating‐content range. Based on this structural verification, LYZnO@NCM and LYZrO@NCM with 0.5 wt% coating were selected as representative samples for detailed surface and microstructural analyses. The surface morphologies and elemental distributions of the representative 0.5 wt% LYZnO@NCM and LYZrO@NCM samples were examined by SEM and EDS elemental mapping. As shown in Figure 2a,b, both coated samples retained the spherical secondary‐particle morphology and densely packed polycrystalline architecture of pristine NCM. No obvious particle collapse, severe surface damage, or morphological reconstruction was observed, indicating that the 0.5 wt% LYZnO and LYZrO coating processes did not significantly affect the overall morphology of the NCM particles. The corresponding EDS elemental mappings revealed homogeneous distributions of Y and Zn in LYZnO@NCM and Y and Zr in LYZrO@NCM over the NCM secondary particles, supporting the successful introduction of LYZnO and LYZrO species through the wet‐coating process. Surface chemical signatures of the coating layers were further verified by XPS analysis, where Y 3d signals were observed for both coated samples, along with Zn 2p signals for LYZnO@NCM and Zr 3d signals for LYZrO@NCM (Figure S7). These results further confirm that the LYZnO and LYZrO species were introduced onto the NCM particle surfaces.

The local microstructures of the LYZnO and LYZrO coating layers were further investigated by high‐resolution transmission electron microscopy (HRTEM) combined with fast Fourier transform (FFT) patterns, as shown in Figure 2c,d. Both LYZnO@NCM and LYZrO@NCM exhibited thin crystalline surface layers (≈10 nm in thickness) closely integrated with the underlying NCM particles (Figure S10). For LYZnO@NCM, a lattice fringe with a d‐spacing of 0.262 nm was observed in the surface region and assigned to the (202) plane of LYZnO, while the underlying NCM region showed a d‐spacing of 0.478 nm corresponding to the (003) plane of layered NCM. Similarly, LYZrO@NCM displayed a d‐spacing of 0.264 nm at the surface, corresponding to the (202) plane of LYZrO, together with the NCM (003) d‐spacing of 0.479 nm [32, 34]. The clear distinction confirms the successful integration of LYZnO and LYZrO coating layers and the corresponding SAED patterns collected from the surface regions further support the crystalline nature of the LYZnO and LYZrO coating layers. These results demonstrate that both LYZnO and LYZrO form uniform nanoscale coating layers on polycrystalline NCM particles without disrupting the host particle morphology. No significant change in the NCM host lattice was observed after the coating process. The refined lattice parameters of LYZnO@NCM and LYZrO@NCM were comparable to those of bare NCM (Figure S8 and Table S3), while XPS depth profiling showed that the Y 3d, Zn 2p, and Zr 3d signals rapidly decreased with increasing etching depth, accompanied by an increase in the Ni 2p intensity (Figure S9). These results suggest that the coating elements are predominantly localized near the particle surface, with no evident incorporation into the bulk NCM lattice. This comparable coating provides a reliable basis for directly evaluating how Li‐excess‐type LYZnO and Li‐vacancy‐type LYZrO influence interfacial stabilization and Li^+^ transport at the NCM–LPSCl interface in ASSBs.

### 2.3 Electrochemical Performance in LIBs

**FIGURE 3:**
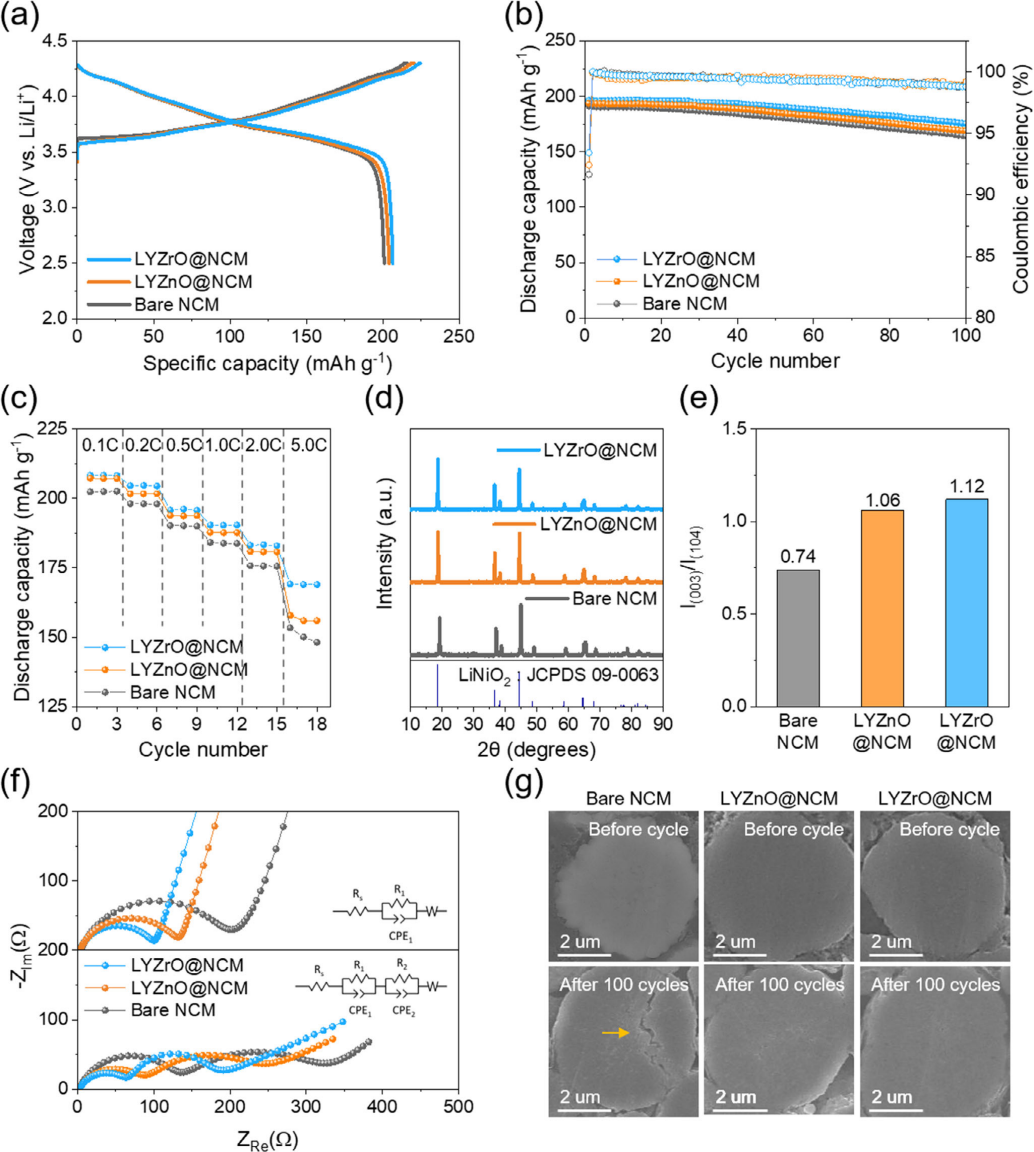
Electrochemical performances of NCM, LYZnO@NCM, and LYZrO@NCM in LIBs; (a) galvanostatic voltage profiles at a current density of 0.1C (1.0C = 200 mA g^−1^) and (b) cycle performances with Coulombic efficiencies at a constant current density of 1.0C in a voltage range of 2.5–4.3 V vs. Li/Li^+^ under room temperature (25°C). (c) rate‐capability performances of NCM, LYZnO@NCM, and LYZrO@NCM at different current densities ranging from 0.1C to 5.0C. (d) XRD patterns of NCM, LYZnO@NCM, and LYZrO@NCM cathodes after 100 cycles in LIBs. (e) Corresponding I_(003)_/I_(104)_ intensity ratios. (f) Nyquist plots after cycling. (g) Cross‐sectional FE‐SEM images of bare NCM, LYZnO@NCM, and LYZrO@NCM cathodes before and after 100 cycles.

The coating amount was further optimized by comparing the initial charge–discharge profiles and cycling performances of LYZnO@NCM and LYZrO@NCM cathodes with coating contents of 0.3, 0.5, and 1.0 wt% in LIB half‐cells (Figure S11). Among the examined coating contents, 0.5 wt% delivered the most balanced electrochemical performance for both LYZnO@NCM and LYZrO@NCM, indicating that an appropriate coating amount is required to provide surface protection without introducing an excessive Li^+^ transport barrier. Therefore, the 0.5 wt% coated samples were used for the representative electrochemical comparison in Figure 3. Using the optimized coating amount, the electrochemical performances of bare NCM, LYZnO@NCM, and LYZrO@NCM were compared in LIB half‐cells employing a liquid electrolyte. This evaluation was conducted to clarify the baseline effect of the LYZnO and LYZrO coating layers on the electrochemical behavior of NCM, independent of possible side reactions with the LPSCl solid electrolyte. As shown in Figure 3a, all cathodes exhibited similar initial charge–discharge voltage profiles at a current density of 0.1C, indicating that the LYZnO and LYZrO coatings did not significantly alter the intrinsic redox behavior of the NCM cathode. Nevertheless, the LYZnO@NCM and LYZrO@NCM cathodes delivered higher initial discharge capacities than bare NCM cathode. In particular, LYZrO@NCM cathode showed the highest discharge capacity of 206.5 mAh g^−1^, compared with 200.9 mAh g^−1^ for bare NCM cathode and 204.1 mAh g^−1^ for LYZnO@NCM cathode. This result suggests that the LYZnO and LYZrO coatings improve the electrochemical utilization of NCM at the first cycle.

The cycling stability was further examined at 1.0C in the voltage range of 2.5–4.3 V at 25°C (Figure 3b). After 100 cycles, the LYZrO@NCM cathode exhibited the highest capacity retention of 87.6%, followed by LYZnO@NCM cathode (87.3%) and bare NCM cathode (85.9%). Although the difference in capacity retention was relatively modest, both LYZnO@NCM and LYZrO@NCM cathodes consistently outperformed bare NCM cathode, suggesting that the LYZnO and LYZrO coating layers effectively mitigate cycling‐induced degradation.

The beneficial role of the LYZnO and LYZrO coatings became more pronounced in the rate‐capability test (Figure 3c). At 5.0C, the LYZrO@NCM cathode retained 81.2% of its capacity at 0.1C, whereas bare NCM cathode retained only 75.8%, with the LYZnO@NCM cathode showing an intermediate value of 76.1%. These results indicate that both LYZnO and LYZrO coatings improve high‐rate performance by facilitating Li^+^ transport across the cathode–electrolyte interface. The superior rate capability of the LYZrO@NCM cathode is consistent with the higher ionic conductivity of LYZrO, suggesting that Li‐vacancy‐type defect chemistry is more effective than Li‐excess‐type defect chemistry in enhancing interfacial Li^+^ transport.

Post‐cycling XRD analysis was conducted after 100 cycles to examine the structural degradation of the cathodes as compared in Figure 3d. Bare NCM showed a pronounced decrease in the I_(003)_/I_(104)_ intensity ratio to 0.74, suggesting increased Li^+^/Ni^2+^ cation mixing and structural disordering during cycling (Figure 3e) [26]. In contrast, LYZnO@NCM and LYZrO@NCM maintained higher I_(003)_/I_(104)_ ratios of 1.06 and 1.12, respectively. These results demonstrate that the LYZnO and LYZrO coatings suppress the irreversible structural degradation of the layered NCM structure by stabilizing the surface of NCM particles.

The interfacial resistance after cycling was evaluated by electrochemical impedance spectroscopy (EIS) (Figure 3f). The charge‐transfer resistance decreased from 180.8 Ω for bare NCM to 150.0 Ω for LYZnO@NCM and 113.6 Ω for LYZrO@NCM. The lower resistance of the LYZnO@NCM and LYZrO@NCM indicates that the LYZnO and LYZrO coating layers suppress impedance growth while maintaining efficient Li^+^ transfer across the cathode–electrolyte interface. Moreover, the lower resistance further supports the beneficial role of Li^+^ defect chemistry of LYO in improving interfacial reaction kinetics.

Cross‐sectional SEM images obtained before and after 100 cycles provided additional evidence for the protective effect of the LYZnO and LYZrO coatings (Figure 3g). After cycling, bare NCM particles exhibited severe intergranular cracking, which can be attributed to anisotropic lattice strain and repeated volume changes during cycling [26]. In contrast, LYZnO@NCM and LYZrO@NCM particles retained more intact secondary‐particle morphologies with substantially reduced internal cracks. This morphological and microstructural preservation suggests that the nanoscale LYZnO and LYZrO coatings alleviate local stress accumulation and suppress electrolyte infiltration into newly formed internal surfaces, thereby reducing mechanical degradation during cycling.

Overall, the results demonstrate that LYZnO and LYZrO coatings improve the electrochemical stability of NCM by enhancing interfacial Li^+^ transport, suppressing impedance growth, and mitigating structural degradation. More importantly, the improvement was consistently greater for LYZrO@NCM than for LYZnO@NCM. This trend indicates that Li‐vacancy‐type defect chemistry is more effective than Li‐excess‐type defect chemistry in stabilizing the NCM particles under LIBs operation with a liquid electrolyte.

### 2.4 Electrochemical Performance in ASSBs

**FIGURE 4:**
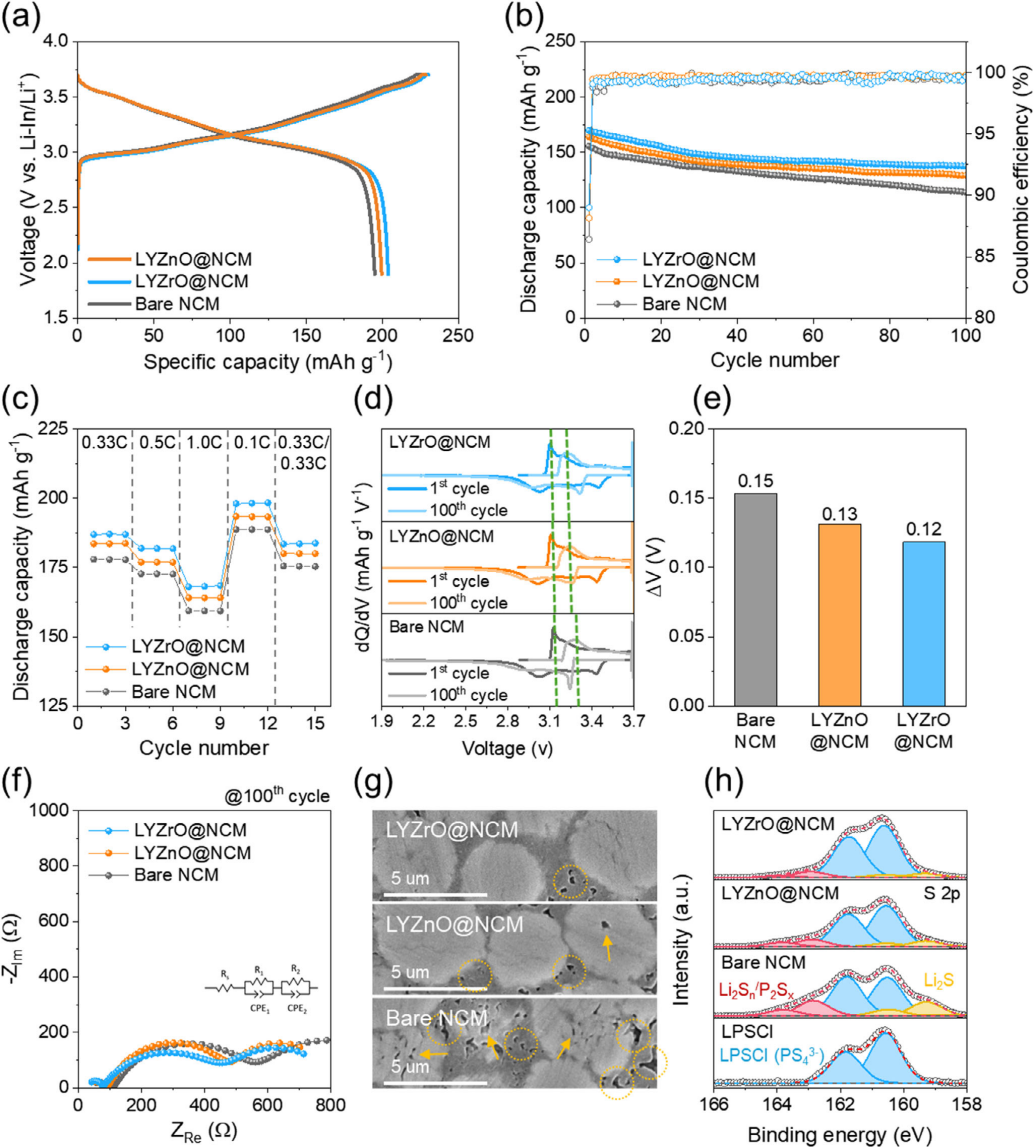
Electrochemical performances of NCM, LYZnO@NCM and LYZrO@NCM in ASSBs; (a) galvanostatic voltage profiles at a current density of 0.1C (1.0C = 200 mA g^−1^) and (b) cycle performances with coulombic efficiencies at a constant current density of 1.0C in a voltage range of 1.9–3.7 V vs. In–Li/Li^+^ under room temperature (25°C). (c) rate‐capability performances of NCM, LYZnO@NCM, and LYZrO@NCM at different current densities ranging from 0.1C to 1.0C. (d) Ex situ dQ/dV plots for the first and 100th cycles at room temperature (25°C). (e) Corresponding voltage shifts obtained from the dQ/dV profiles. (f) Nyquist plots and (g) Cross‐sectional FE‐SEM images and (h) XPS spectra of bare NCM, LYZnO@NCM, and LYZrO@NCM after 100 cycles in ASSBs.

The cathodes were further evaluated in ASSBs to verify whether the LYZnO and LYZrO coatings could stabilize the NCM–LPSCl interface. As shown in the initial charge–discharge profiles measured at 0.1C (Figure 4a), both LYZnO@NCM and LYZrO@NCM cathodes exhibited improved initial electrochemical behaviors compared with bare NCM cathode. As expected, the LYZrO@NCM cathode delivered the highest initial discharge capacity of 203.7 mAh g^−1^ and the highest initial coulombic efficiency of 88.4%, which were higher than those of bare NCM cathode (195.0 mAh g^−1^ and 86.3%). The LYZnO@NCM cathode showed intermediate values of 199.5 mAh g^−1^ and 86.3%. To examine the capacity contribution of the coating materials, LYZnO and LYZrO were independently evaluated in ASSB half‐cells. Both materials showed negligible capacities and no distinct redox peaks within the same voltage range (Figure S12), indicating that the coating layers make a negligible contribution to the measured capacity. These results indicate that LYZnO and LYZrO coating layers effectively suppress irreversible interfacial reactions during the first cycle by reducing direct contact between NCM and LPSCl.

The long‐term cycling performance was examined at 1.0C within 1.9–3.7 V vs. In–Li/Li^+^ (Figure 4b). After 100 cycles, LYZrO@NCM cathode retained 81.2% of its initial capacity, outperforming both LYZnO@NCM cathode (78.7%) and bare NCM cathode (73.2%). The improvement in capacity retention was more pronounced in ASSBs than in LIBs, suggesting that NCM–LPSCl interfacial degradation is a major origin of capacity fading in ASSBs. The rate‐capability tests further support this interpretation as presented in Figure 4c. Both the LYZnO@NCM and LYZrO@NCM cathodes maintained higher discharge capacities than bare NCM cathode over the current‐density range and showed higher capacity retentions even at 1.0C. This result indicates that the LYZnO and LYZrO coatings do not act as severe Li^+^ transport barriers within the composite cathode of ASSBs. The superior rate capability of the LYZrO@NCM cathode is consistent with the higher ionic conductivity enabled by its Li‐vacancy‐type defect chemistry.

Differential capacity (dQ/dV) profiles are compared to evaluate polarization growth during cycling (Figure 4d,e). From the first to the 100^th^ cycle, bare NCM cathode exhibited a redox‐peak voltage shift of 0.15 V, whereas LYZrO@NCM cathode showed a smaller shift of 0.12 V and LYZnO@NCM displayed an intermediate voltage shift of 0.13 V. The reduced voltage shifts of the coated cathodes indicate that the LYZnO and LYZrO layers effectively alleviate the progressive polarization during cycling. This behavior is consistent with the mitigated deterioration of interfacial reaction kinetics at the NCM–LPSCl interface during cycling.

Post‐cycling EIS analysis further confirmed the reduced interfacial resistance of the LYZnO@NCM and LYZrO@NCM cathodes (Figure 4f). After 100 cycles, the charge‐transfer resistance decreased from 277.2 Ω for the bare NCM cathode to 227.8 Ω for the LYZnO@NCM cathode and 205.5 Ω for the LYZrO@NCM cathode. The lower resistance can be attributed to the suppressed formation of resistive interphase products and the preservation of Li^+^ transport pathways across the NCM–LPSCl interface. Temperature‐dependent EIS measured at a fixed state of charge after formation showed that the activation energy associated with the interfacial process decreased from 0.50 eV for bare NCM to 0.43 eV for LYZnO@NCM and 0.42 eV for LYZrO@NCM (Figure S13), providing a quantitative kinetic parameter that complements the rate‐capability and post‐cycling resistance data. DRT analysis further supported the reduced interfacial resistance of the coated cathodes.

The improved interfacial stability was also supported by post‐cycling cross‐sectional SEM analysis (Figure 4g). Cross‐sections of the pristine electrodes showed comparable particle packing and interfacial contact among the three cathodes (Figure S14), suggesting that the differences observed after cycling mainly developed during cycling. After 100 cycles, the bare NCM cathode exhibited severe morphological degradation, including pronounced void formation and contact loss at the NCM–LPSCl interface. Such interfacial deterioration can disrupt continuous Li^+^ transport pathways and accelerate impedance growth during cycling. In contrast, both LYZnO@NCM and LYZrO@NCM cathodes maintained more continuous interfacial contact with fewer voids. This morphological preservation indicates that the LYZnO and LYZrO coatings alleviate contact loss by suppressing direct chemical and electrochemical reactions between NCM and LPSCl.

Post‐cycling S 2p XPS analysis provided direct chemical evidence for the suppressed decomposition of the LPSCl in ASSBs (Figure 4h). Pristine LPSCl exhibited the characteristic thiophosphate signal associated with PS_4_^3−^ units, whereas the cycled bare NCM cathode showed pronounced signals for decomposition products, assigned to Li_2_S_n_/P_2_S_x_ species near 162 eV and Li_2_S near 160 eV [14, 29]. These species indicate the oxidative decomposition of LPSCl at the high‐voltage operation. In contrast, both the LYZnO@NCM and LYZrO@NCM cathodes showed markedly weaker signals, while the PS_4_^3−^ contribution remained dominant. This result demonstrates that the LYZnO and LYZrO coatings suppress the accumulation of resistive sulfide‐derived interphase products by limiting direct NCM–LPSCl contact while maintaining Li^+^ transport across the interface.

Overall, we note that LYZnO and LYZrO coatings improve the electrochemical stability of NCM cathodes by suppressing LPSCl decomposition, reducing interfacial resistance, mitigating polarization growth, and preserving NCM–LPSCl contact. More importantly, the extent of improvement was consistently greater for Li‐vacancy‐type LYZrO@NCM than for Li‐excess‐type LYZnO@NCM in ASSBs.

## 3 Conclusions

This study demonstrates that Li^+^ point‐defect chemistry plays a decisive role in the design of ion‐conductive coatings for NCM cathodes. Li‐excess‐type LYZnO and Li‐vacancy‐type LYZrO were rationally designed within the same monoclinic LiYO_2_ framework, enabling a direct comparison of how different Li^+^ point defect chemistries affect Li^+^ transport and interfacial stabilization. Notably, the Li‐vacancy‐type LYZrO delivered higher ionic conductivity and consistently superior stabilization effects compared to Li‐excess‐type LYZnO. In ASSBs, LYZrO@NCM effectively mitigated polarization growth, reduced charge‐transfer resistance, preserved interfacial contact, and suppressed the formation of sulfide‐derived decomposition products. These findings confirm that Li‐vacancy engineering is a more effective strategy than Li‐excess‐type defect formation for constructing stable and ion‐conductive interlayers. Ultimately, this work opens up a new strategic pathway for defect‐engineered coating materials aimed at improving the structural and interfacial stability of NCM cathodes in ASSBs.

## 4 Experimental Section

### 4.1 Material Preparation

LiYO_2_ (LYO), Li_1.05_Y_0.95_Zn_0.05_O_2_ (LYZnO), and Li_0.95_Y_0.95_Zr_0.05_O_2_ (LYZrO) powders were synthesized via a sol‐gel‐based wet chemical route. Yttrium acetate tetrahydrate ((CH_3_CO_2_)_3_Y·4H_2_O, >99%, Sigma‐Aldrich), lithium hydroxide (LiOH, >98%, Sigma‐Aldrich), and citric acid (C_6_H_8_O_7_, >99.5%, Sigma‐Aldrich) were dissolved in 20 mL of ethanol at a molar ratio of 1:1:0.8 and stirred at 80°C and 300 rpm for 1 h. For LYZnO synthesis, zinc acetate (Zn(CH_3_COO)_2_·2H_2_O, >99%, Sigma‐Aldrich) was incorporated alongside the yttrium precursor at the appropriate molar ratio, while for LYZrO synthesis, zirconium acetate (Zr(CH_3_COO)_4_, >99%, Sigma‐Aldrich) was used accordingly. The amount of the Li precursor was adjusted in each case to match the target stoichiometry (Li excess for LYZnO; Li vacancy for LYZrO). After solvent evaporation, the resulting powder was finely ground and calcined at 700°C for 4 h under a flowing O_2_ atmosphere to yield the final product.

For the preparation of LYZrO@NCM composite powder, the LYZrO precursor solution was prepared at the molar ratio corresponding to the target coating amount following the same procedure described above. Commercial NCM (LiNi_0.82_Co_0.15_Mn_0.03_O_2_) powder was added to the precursor solution and stirred at 80°C and 300 rpm for 1 h to ensure uniform mixing. After drying, the powder was calcined at 700°C for 4 h under O_2_ to obtain LYZrO@NCM powder. LYZnO@NCM was prepared under identical conditions.

### 4.2 Structural Characterizations

The morphological and microstructural features of the samples were examined using field‐emission scanning electron microscopy (FESEM, JSM‐7000F, JEOL), transmission electron microscopy (TEM, JEM‐3010, JEOL), and energy‐dispersive X‐ray spectroscopy (EDS). Crystal structures were characterized by powder X‐ray diffraction (XRD, Empyrean, PANalytical) and Raman spectroscopy (BX43, OLYMPUS). Surface chemical states were analyzed by X‐ray photoelectron spectroscopy (XPS, K‐alpha, Thermo Scientific). The elemental compositions of Li, Y, Zn, and Zr in each sample were quantitatively determined by inductively coupled plasma mass spectrometry (ICP‐MS, Agilent 7500cx, Agilent).

LYZnO/LPSCl and LYZrO/LPSCl mixtures (50:50 wt%) were aged at 80°C for 24 h under Ar and subsequently analyzed by XRD and XPS without air exposure.

### 4.3 Electrochemical Measurements

Ionic conductivity was measured using a symmetric cell configuration (solid electrolyte | sample | solid electrolyte). EIS measurements on symmetric cells were conducted over a frequency range (7 MHz to 0.1 Hz) with an amplitude of 10 mV. Resistance (R) was determined from Nyquist plots, and ionic conductivity (σ) was calculated as follows:

σ=L/(R×A)

where *L* and *A* were the sample thickness and electrode area, respectively. Linear sweep voltammetry (LSV) was performed using a potentiostat (Bio‐Logic, VSP‐300) over a voltage range of 2.5–5.0 V at a scan rate of 0.1 mV s^−^^1^. Temperature‐dependent EIS measurements were conducted from 20 to 60°C at 10°C intervals. Activation energies were determined from Arrhenius plots of ln(σT) versus 1000/T, and pellet relative densities were calculated using the geometric and crystallographic densities.

For LIB performance evaluation, CR2032 coin‐type half‐cells were assembled. Cathode slurries were prepared by mixing the active material (NCM or coated samples), conducting agent (Super‐C), and binder (polyvinylidene fluoride, PVdF) at a weight ratio of 94:3:3 in N‐methyl‐2‐pyrrolidone (NMP) solvent, and cast onto Al foils (15 µm thick). The electrodes were initially dried in a convection oven at 80°C for 1 h. Roll‐pressing was then applied to achieve a loading level of 11.0 mg cm^−2^ and an electrode density of 3.2 g cm^−3^. Subsequently, they were further dried at 120°C for 10 h under vacuum prior to cell assembly. Half‐cells were fabricated in an Ar‐filled glove box using lithium metal foil (200 µm) as the counter electrode and an electrolyte comprising 1 M LiPF_6_ dissolved in ethylene carbonate (EC) and dimethyl carbonate (DMC) (3:7, v/v). Electrochemical performance was evaluated using a battery cycler (Won‐A Tech, WBCS3000L) at current densities ranging from 0.1C to 5.0C (1.0C = 200 mA g^−1^) within a voltage range of 2.5–4.3 V vs. Li/Li^+^ at 25°C. Electrochemical impedance spectroscopy (EIS) was conducted over a frequency range of 1.0 MHz to 0.5 mHz with an excitation amplitude of 5.0 mV using a potentiostat (Bio‐Logic, VSP‐300).

ASSB cells were fabricated in a controlled dry‐room environment. The cathode composite was prepared by blending NCM (active material), Li_6_PS_5_Cl (solid electrolyte), and Super P (conducting agent) in a weight ratio of 80:19:1. Solid electrolyte pellets were produced by compressing 100 mg of Li_6_PS_5_Cl powder under a uniaxial pressure of 120 MPa (diameter < 10 mm). The pellet was then stacked with approximately 10 mg of the cathode composite and Li‐In alloy foil. The multilayer configuration (Al foil | cathode composite | Li_6_PS_5_Cl | Li–In | SUS foil) was pressed under 170 MPa, and cells were sealed with a tightening torque of 11 N·m. Electrochemical performance was evaluated using a battery cycler (TOSCAT‐3100, TOYO) at current densities ranging from 0.1C to 1.0C (1.0C = 200 mA g^−1^) within a voltage range of 1.9–3.7 V vs. In–Li/Li^+^ at 25°C. EIS was performed using a BioLogic VSP‐300 analyzer over a frequency range of 1.0 MHz to 0.5 mHz with an excitation amplitude of 5 mV. LYZnO‐ and LYZrO‐based ASSB half‐cells were evaluated over 1.9–3.7 V vs. In–Li/Li^+^ by galvanostatic cycling and cyclic voltammetry. Temperature‐dependent EIS measurements were conducted from 25 to 60°C at a fixed state of charge, and the apparent interfacial activation energy was obtained from the DRT‐resolved R*int*.

## Author Contributions

**Sodam Kim**: writing – original draft, methodology, visualization, investigation, conceptualization. **Yun Seong Byeon**: methodology, data curation, investigation, visualization. **Jaewoo Jung**: formal analysis, methodology. **Mingyu Cho**: methodology, software. **Hyunmin Lee**: methodology, software. **Jung Ho Kim**: writing – review and editing, supervision, methodology, validation. **Min‐Sik Park**: writing – review and editing, funding acquisition, project administration, conceptualization.

## Funding

National Research Foundation of Korea (NRF) grant funded by the Korea government (MSIT) (No. RS‐2024‐00450728 and RS‐2025‐25441255).

## Conflicts of Interest

The authors declare no conflicts of interest.

## Supporting information




**Supporting File**: advs77729‐sup‐0001‐SuppMat.docx.

## Data Availability

The data that support the findings of this study are available in the supporting information of this article.
