# Supplementary material for: Defect‐Engineered LiYO2 Interlayers Enabling Fast Interfacial Li+ Transport in All‐Solid‐State Batteries
Source: Adv Sci (Weinh). 2026 Sep 10:e77729. Online ahead of print. doi: 10.1002/advs.77729 (PMC13562772; doi:10.1002/advs.77729)
Supplement: Supplementary file 1 [file ADVS-9999-e77729-s001.docx]

Supporting Information

Defect-Engineered LiYO_2_ Interlayers Enabling Fast Interfacial Li^+^ Transport in All-Solid-State Batteries

Sodam Kim^a^, Yun Seong Byeon^a^, Jaewoo Jung^a^, Mingyu Cho^a^, Hyunmin Lee^a^, Jung Ho Kim^b,*^ and Min-Sik Park ^a,*^

S. Kim, Y. S. Byeon, J. Jung, M. Cho, H. Lee, M.-S. Park

Department of Materials Science and Engineering, Kyung Hee University, 1732 Deogyeong-daero, Giheung-gu, Yongin 17104, Republic of Korea
E-mail: mspark@khu.ac.kr

J. H. Kim
Institute for Superconducting & Electronic Materials (ISEM), Australian Institute for Innovative Materials, University of Wollongong, North Wollongong, NSW, Australia
E-mail : jhk@uow.edu.au

**Figure S1.** Cationic elemental composition (atomic percent, normalized to total cation content) of the LYO, LYZnO, and LYZrO powders determined by inductively coupled plasma mass spectrometry (ICP-MS).

**Figure S2.** Rietveld refinement profiles of (a) LYO, (b) LYZnO, and (c) LYZrO powders, showing the observed (I_obs_), calculated (I_cal_), and difference patterns.

**Table S1**. Refined lattice parameters, unit-cell volumes, fixed site occupancy factors, and agreement factors of LYO, LYZnO, and LYZrO obtained from occupancy-constrained Rietveld refinement.

Supplementary Note 1. Occupancy-constrained Rietveld refinement of the LYO-based coating materials

Because Li has a substantially lower X-ray scattering factor than Y, reliable refinement of the Li-site occupancy from laboratory Cu Kα XRD is difficult and strongly correlated with other refinement parameters. Therefore, rather than freely refining the Li occupancy, the cation occupancies were constrained using the compositions determined by ICP-MS (Figure S1) where they could be represented within the crystallographic model. For LYZnO, the regular Li site was fixed at full occupancy (SOF = 1.00) because a crystallographic site cannot exceed unity, while the excess Li detected by ICP-MS was not assigned to an additional crystallographic site because its position could not be reliably resolved from the laboratory XRD data. The refinements were intended to evaluate whether the ICP-derived compositions were structurally consistent with the measured diffraction profiles rather than to independently quantify the Li content. The monoclinic LiYO_2_ structure (space group P2_1_/c, Materials Project mp-7020) was used as the starting model, and the scale factor, background, specimen displacement, lattice parameters, peak-shape parameters, and preferred orientation were sequentially refined while the atomic coordinates and displacement parameters were fixed. The resulting lattice parameters and agreement factors are summarized in Table S1.

The refinements yielded comparable R_wp_ values of 8.29–9.39% for LYO, LYZnO, and LYZrO. Relative to pristine LYO, the unit-cell volume decreased by 0.35% for LYZnO and increased by 0.79% for LYZrO. Because Zn^2+^ and Zr^4+^ have similar ionic radii, these opposite volume changes cannot be explained solely by the size of the substituent cations and are consistent with different responses of the Li sublattice induced by aliovalent substitution. Zn^2+^ substitution is associated with Li-excess-type defect chemistry, whereas Zr^4+^ substitution is associated with Li-deficient defect chemistry. These structural trends therefore support, but do not independently prove, the proposed defect models.

No secondary crystalline phase was detected in the diffraction patterns. In addition, pristine LYO synthesized under identical conditions exhibited a nearly stoichiometric Li/Y ratio of 49.98/50.02 at% by ICP-MS. These observations argue against significant Li volatilization arising solely from the calcination process, although minor composition-specific Li loss cannot be completely excluded. Because laboratory XRD has limited sensitivity to the Li sublattice, the present refinement does not constitute a direct measurement of Li vacancies. Instead, the proposed defect chemistry is supported by the combined ICP-MS compositions, occupancy-constrained refinement, lattice-volume evolution, and complementary spectroscopic results.

**Figure S3.** Raman spectra of LiYO_2_, Li_1.05_Y_0.95_Zn_0.05_O_2_, and Li_0.95_Y_0.95_Zr_0.05_O_2_ powders.

**Table S2.** Pellet thickness, relative density, total resistance, and room-temperature ionic conductivity of the LYO, LYZnO, and LYZrO coating materials, determined by electrochemical impedance spectroscopy (EIS) on pressed pellets with ion-blocking electrodes.

**Figure S4.** (a) Nyquist plots of LYO, LYZnO, and LYZrO pellets measured with ion-blocking electrodes at 20, 30, 40, 50, and 60 °C, with magnified high-frequency regions shown as insets. (b) Corresponding Arrhenius plots of ln(σT) versus 1000/T and (c) the activation energies for ionic conduction obtained from the linear fits.

**Figure S5.** (a) XRD patterns of LPSCl, LYZnO–LPSCl, and LYZrO–LPSCl with reference patterns of Li_7_PS_6_ (JCPDS 34-0688) and LiYO_2_ (JCPDS 24-0671). (b) Magnified 25–30° region of pristine LPSCl and the LYZnO–LPSCl and LYZrO–LPSCl mixtures after storage at 80 °C for 24 h, with the Li_2_S region highlighted. (c) S 2p and (d) P 2p XPS spectra of the same samples.

**Figure S6.** (a,b) SEM images with corresponding EDS elemental mapping results and (c,d) powder XRD patterns of (a,c) LYZnO@NCM and (a,d) LYZrO@NCM with different coating amounts (0.3, 0.5, and 1.0 wt%).

**Figure S7.** XPS spectra of bare NCM, LYZnO@NCM, and LYZrO@NCM in the (left) Y 3d, (middle) Zr 3d, and (right) Zn 2p regions.

**Figure S8**. (a) XRD patterns, (b) (003) interplanar spacings, and (c) refined lattice parameters (a, c, and c/a) of bare NCM, LYZnO@NCM, and LYZrO@NCM.

**Table S3**. Refined lattice parameters, c/a ratios, unit-cell volumes, and agreement factors of bare NCM, LYZnO@NCM, and LYZrO@NCM.

**Figure S9.** XPS depth profiles of (a) bare NCM, (b) LYZnO@NCM, and (c) LYZrO@NCM in the Ni 2p, Y 3d, Zn 2p, and Zr 3d regions.

**Figure S10.** TEM images with corresponding EDS elemental mapping results of LYZnO@NCM and LYZrO@NCM.

**Figure S11.** (a,b) Galvanostatic voltage profiles and (c,d) cycle performances of bare NCM, LYZnO@NCM, and LYZrO@NCM with various concentrations (0.3, 0.5, and 1.0 wt%) in LIBs.

**Figure S12**. (a) Initial galvanostatic voltage profiles of ASSB half-cells employing LYZnO and LYZrO as the sole active materials. (b) Voltage responses over two consecutive cycles versus elapsed time. (c) Cyclic voltammograms of the LYZnO and LYZrO half-cells measured over 1.9–3.7 V vs. In–Li/Li^+^ at a scan rate of 0.1 mV s^−1^.

**Figure S13**. (a) Nyquist plots of bare NCM, LYZnO@NCM, and LYZrO@NCM ASSB half-cells measured at 25, 30, 40, 50, and 60 °C at a fixed state of charge after formation. (b) Corresponding Arrhenius plots of ln(T/R_int_) versus 1000/T and (c) the activation energies for interfacial Li^+^ transport. (d) DRT spectra of the bare NCM, LYZnO@NCM, and LYZrO@NCM ASSB half-cells.

**Figure S14.** Cross-sectional FE-SEM images of the pristine (a) bare NCM, (b) LYZnO@NCM, and (c) LYZrO@NCM composite electrodes prepared by cross-section polishing.
